# Molecular Identification of Prune Dwarf Virus (PDV) Infecting Sweet Cherry in Canada and Development of a PDV Full-Length Infectious cDNA Clone

**DOI:** 10.3390/v13102025

**Published:** 2021-10-07

**Authors:** Aaron J. Simkovich, Yinzi Li, Susanne E. Kohalmi, Jonathan S. Griffiths, Aiming Wang

**Affiliations:** 1 London Research and Development Centre, Agriculture and Agri-Food Canada, 1391 Sandford Street, London, ON N5V 4T3, Canada; asimkovi@uwo.ca (A.J.S.); Yinzi.Li@agr.gc.ca (Y.L.); 2 Department of Biology, The University of Western Ontario, 1151 Richmond Street, London, ON N6A 5B7, Canada; skohalmi@uwo.ca; 3 London Research and Development Centre, Agriculture and Agri-Food Canada, 4902 Victoria Ave N, Vineland Station, ON L0R 2E0, Canada; Jonathan.Griffiths@agr.gc.ca

**Keywords:** fruit tree virus, *Prune dwarf virus*, ilarvirus, infectious clone, next generation sequencing

## Abstract

*Prune dwarf virus* (PDV) is a member of ilarviruses that infects stone fruit species such as cherry, plum and peach, and ornamentally grown trees worldwide. The virus lacks an RNA silencing suppressor. Infection by PDV either alone, or its mixed infection with other viruses causes deteriorated fruit marketability and reduced fruit yields. Here, we report the molecular identification of PDV from sweet cherry in the prominent fruit growing region of Ontario, Canada known as the Niagara fruit belt using next generation sequencing of small interfering RNAs (siRNAs). We assessed its incidence in an experimental farm and determined the full genome sequence of this PDV isolate. We further constructed an infectious cDNA clone. Inoculation of the natural host cherry with this clone induced a dwarfing phenotype. We also examined its infectivity on several common experimental hosts. We found that it was infectious on cucurbits (cucumber and squash) with clear symptoms and *Nicotiana benthamiana* without causing noticeable symptoms, and it was unable to infect *Arabidopsis thaliana*. As generating infectious clones for woody plants is very challenging with limited success, the PDV infectious clone developed from this study will be a useful tool to facilitate molecular studies on PDV and related *Prunus*-infecting viruses.

## 1. Introduction

**Figure 1:**
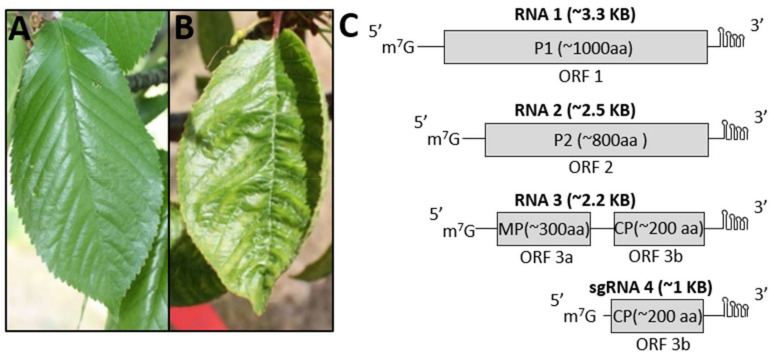
Source and genome structure of PDV. (**A**) A healthy, asymptomatic leaf from a cherry tree showing an even distribution of green colouring, absence of damage or other deformations. (**B**) A symptomatic leaf from a cherry tree showing symptoms commonly associated with viral infection including chlorosis (yellowing) and uneven distribution of green colouring, vein suturing and leaf curl. (**C**) A diagram showing the genome structure of PDV. Every genomic ssRNA( + ) fragment is shown with approximate lengths in parentheses. Encoded proteins are shown as gray boxes. All RNA fragments have putative m7G cap structures at the 5′ UTR, and each 3′ UTR is predicted to adopt complex secondary structures. RNA 1 encodes the P1 protein which has Met and Hel domains which are essential for viral replication. RNA 2 encodes the RdRp (P2). RNA 3 directly encodes the MP. The fourth RNA fragment, sgRNA 4 is transcribed from RNA 3 downstream of ORF3a and encodes the viral CP. m7G: 5′-7-methyl-G cap; 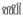:3′ UTR secondary structure; aa: amino acids; bp: base pair; P1: replicase protein; P2: RNA dependent RNA polymerase (RdRp); MP: movement protein; CP: coat protein.

The genus *Prunus* consists of many important fruit species such as peach, cherry, plum, apricot and nectarine as well as a variety of ornamental crops. Like other perennials, these *Prunus* species are affected by numerous viruses [1,2]. Among them, *Prune dwarf virus* (PDV) is an important pathogen, known to infect all members of the *Prunus* genus, impacting both stone fruit, and ornamental crop production [3]. The virus is widespread in stone fruit growing regions globally and is known to transmit via seed and pollen and during plant propagation practices such as grafting [4,5,6]. PDV is a member of the *Ilarvirus* genus within the family *Bromoviridae*. Currently, ilarviruses have been phylogenetically classified into four subgroups and all members of subgroups 3 and 4 lack an RNA silencing suppressor [7]. PDV belongs to subgroup 4. Virions of PDV are *quasi*-isometric in shape, ranging in diameter from 26–35 nm [4]. This virus has a tripartite genome of single-stranded positive sense RNA (ssRNA +) (Figure 1). Each viral RNA fragment is separately encapsulated as a virion [7]. The genomic RNAs have a 5′ 7-methyl-G (m7G) capped untranslated region (UTR) and lack a polyadenylated (poly-A) tail at the 3′ end [3,4,8]. Instead, the 3′ UTRs of these genomic RNAs contain repeats of nucleotides AUG and C that are predicted to flank the bases of that form complex secondary RNA structures essential for the regulation of ilarvirus replication and protein translation [9,10]. Among the 3 genomic RNAs, RNA1 is the largest genomic RNA containing a single open reading frame (ORF), ORF1, which encodes the P1 protein. This protein has a methyltransferase (Met) domain near the N-terminus and a helicase (Hel) domain near the C-terminus. The second largest genomic RNA, RNA2, contains ORF2a, encoding the P2 protein, which is an RNA dependent RNA polymerase (RdRp). Together, P1 and P2 form the viral replicase complex [8]. RNA3, is bicistronic, containing ORF3a and ORF3b. ORF3a encodes the movement protein (MP) which is directly translated from RNA3. A putative subgenomic (sg) promoter region downstream of ORF3a allows for the transcription of a sgRNA fragment (sgRNA4), encoding ORF3b from which the viral coat protein (CP) is translated [8].

PDV infection is associated with dwarfed phenotypes of infected trees, and reduced yields [11,12]. The virus is often found to co-infect trees with other viruses. When co-infected with *Prunus necrotic ringspot virus* (PNRSV), another ilarvirus, the trees develop stronger symptoms compared to infection by PDV or PNRSV alone, leading to a disease syndrome known as “peach stunt” [12]. Despite the great geographic distribution of PDV and the severity of disease complexes associated with this virus, PDV is understudied, especially in the areas such as PDV pathogenesis, synergistic interactions with other viruses in mixed infections and molecular PDV–host interactions [4].

Infectious cDNA clones of plant viruses are extremely powerful tools for studying RNA virus pathogenicity, and are essential for reverse genetic studies of these pathogens [13]. Indeed, many infectious clones for other plant viruses have been developed, and their utility to further our understanding of processes such as virus movement has been shown [14,15]. Additional uses for infectious clones include the in planta production of epitopes against viral pathogens of livestock [16]. Unfortunately, construction of infectious clones for woody plant-infecting viruses has only achieved very limited success and there are very few viral infectious clones for woody plants available [17]. To date, none has been reported for PDV.

In this study, we conducted a high throughout sequencing of small RNAs isolated from leaf samples of sweet cherry (*Prunus avium* L. cv ‘Vista’) grown in Ontario, Canada, and detected the presence of PDV. We further determined its complete genome sequence and developed a PDV full-length infectious cDNA clone. The clone can efficiently infect cherry, resulting in the dwarfed phenotype. In addition, we tested several commonly used model herbaceous plants for PDV infectivity and found that the infectious clone is infectious on cucumber (*Cucumis sativus* L. cv ‘Wisconsin’) and the infection causes typical viral symptoms. Therefore, cucumber may serve as a suitable model plant for the characterization of PDV–plant interactions.

## 2. Materials and Methods

### 2.1. Plant Materials

Asymptomatic or symptomatic leaves (Figure 1) were collected from cherry trees (cv ‘Vista’) grown on the Vineland research farm in Jordan, Ontario, during the months of June and July of 2014–2018. To adequately sample an orchard grown tree, 4 newly emerging leaves were collected from three branches (a total of 12 leaves) and pooled together. The pooled leaf samples were flash frozen in liquid nitrogen and stored at −80°C until future use. To determine the in-field incidence of PDV infection, all 92 trees in a plot were sampled for double antibody sandwich enzyme linked immunosorbent assay (DAS-ELISA) and half of the trees (every other tree) were sampled and processed for total RNA isolation and subsequent RT-PCR analyses. 

Sweet cherry ‘Vista’ seedlings were also used to test the infectivity of the PDV clone. In brief, seeds from cherry trees were cleaned using the following protocol. The mesocarp of fully ripened drupes were mechanically removed from the endocarp and were cleaned and then sterilized in a seed sterilizing solution (1% Maestro 80DF, 0.03% Tween-20) for 1 h and then dried at room temperature. Seeds were cold stratified by storing the cleaned seeds at 4 °C in darkness for 3 months. The cleaned pits were then cracked with a nutcracker to remove the hard endocarp and expose the seed. Shell-free seeds were sterilized with 70% ethanol and 20% commercial bleach, then rinsed with sterile water several times. The surface-sterilized seed were soaked in sterile water for 2 days to allow the seeds to become imbibed and the seed coat was manually removed with forceps. The cleaned, uncoated seeds were then sown in a 1:1 mixture of Promix BX and perlite in 3-inch nursery pots in a greenhouse, a heated seed germination mat was placed under the pots to promote germination.

Additionally, other plants commonly used as experimental hosts in fruit tree virus research were used for infectivity studies including cucumber, squash (*Cucurbita maxima* L. cv ‘buttercup’), tobacco (*Nicotiana benthamiana*) and Arabidopsis (*Arabidopsis thaliana*, Columbia zero ecotype). All plant seedlings were maintained in a growth chamber with a 16 h photoperiod at 25 °C and 8 h of darkness at 20 °C.

### 2.2. Small RNA Extraction and NGS

Small RNAs (sRNAs) were isolated from symptomatic tissues using the mirPremier microRNA isolation kit (Sigma Aldrich Canada, Oakville, ON, Canada) according to manufacturer’s specifications. Isolated sRNAs were used to construct two libraries using the TruSeq Small RNA Sample Prep Kit and sequenced using the MiSeq Desktop Sequencer utilizing the MiSeq v2 reagent 50 cycle PE kit (Illumina, San Diego, CA, USA). After sequencing, Trimmomatic [18] was used to remove the adapter sequences, the reads were then assembled into contigs using Velvet Assembler 0.7.31 [19] and Oases 0.2.09 with k-mers of 17. The assembled contigs were then searched against the NCBI nucleotide database using the BLASTn search tool [20].

### 2.3. RNA Extraction, cDNA Synthesis, and Sequencing of the Genomic RNAs of PDV

Leaves on cherry trees from the same orchard plot showing mottle and vein suturing symptoms were also used as a source of total RNAs. Total RNA was isolated from these tissues following methods described previously [21]. In brief, 100 mg of leaf tissues were homogenized and resuspended in RNA extraction buffer (2% cetyl trimethylammonium bromide, 20 mM ethylenediaminetetraacetic acid, pH 8, 1.4 M NaCl, 100 mM Tris-HCl pH 8, 20% PVP-40). Total RNA was isolated by precipitation with 4 M LiCl and was resuspended in RNAse free water before cDNA synthesis, which was performed using the superscript III cDNA synthesis kit (Thermo Fisher Scientific, Mississauga, ON, Canada).

To verify the full genome sequence of PDV obtained by small RNA sequencing, 5′ and 3′ termini were amplified and sequenced using 5′ and 3′ rapid amplification of cDNA ends (RACE) kits (Thermo Fisher Scientific, Mississauga, ON, Canada) and primers specific to the sequences of each PDV genomic fragment (Supplementary Table S1). Once the 5′ and 3′ ends of each genomic RNA was determined, primers were designed to amplify the full-length genomic RNAs (Supplementary Table S1). All amplification steps were performed using the Phusion high fidelity DNA polymerase (New England Biolabs, Whitby, ON, Canada). PCR reactions were comprised of 1 μL of newly synthesized cDNAs, 1μL of each forward and reverse primer (each at a concentration of 0.2 μM), 0.5 μL of polymerase enzyme, 10 μL of 5× Phusion HF buffer, 1 μL of DNTPs (each at a concentration of 25 mM) and 35.5 μL of sterile distilled water. PCR was performed using an initial denaturation step of 30 s at 98 °C, followed by 35 cycles of 30 s at 98 °C for denaturation, annealing for 30 s at 54 °C, and 2 min at 72 °C for extension, followed by a 5 min extension step at 72 °C. The amplified genomic fragments were then cloned into the pCR^TM^-Blunt cloning vector (Thermo Fisher Scientific, Mississauga, ON, Canada) and sequenced using the Sanger method (Eurofins Genomics, Louisville, KY, USA).

### 2.4. Construction of the Infectious cDNA Clone

The binary vector pCB-Rz was used as the plasmid backbone of the PDV infectious clone. This vector was constructed recently [16] and contains a double *35S* promoter and a ribozyme sequence upstream of the terminator. The full-length cDNA sequences corresponding to the three genomic RNAs were amplified by PCR using primers which contained identical sequences to the viral RNA, and the backbone vector sequences. pCB-Rz was amplified using primers which shared sequence identity with the vector and the 5′ and 3′ termini of the viral RNA fragments (Supplementary Table S1). The sequence identity of the amplified viral cDNAs and the vector allowed for the facile cloning of three amplified RNA fragments which were separately cloned into the amplified PCB-Rz vector using the in-fusion cloning system (Takara Bio, Ann Arbor, MI, USA). Constructs containing the single viral cDNAs were named pPDV1-301, pPDV2-301 and pPDV3-301 to indicate the presence of RNA1, RNA2 or RNA3 within the PCB-Rz vector. The complete sequence of each clone was confirmed via Sanger sequencing and primer walking (Supplementary Table S1).

### 2.5. Combining cDNAs of RNA1 and RNA2

Previous work illustrated that the combination of the cDNAs of RNA1 and RNA2 into a single construct significantly increased the infectivity of a PNRSV infectious clone [22]. We utilized a similar strategy to increase the infectivity of the PDV infectious clone. Sequence analysis revealed the presence of a single *Nar*I restriction enzyme site in the PCB-Rz vector, which was absent in both RNA1 and RNA2 sequences of PDV. The cDNA fragment containing the full length RNA2 and its transcription regulatory elements was amplified from pPDV2-301 using specially designed primers containing flanking *Nar*I sites (Supplementary Table S1). The resulting amplicon was digested with *Nar*I and ligated into the backbone of pPDV1-301 digested with *Nar*I. The construct containing both cDNAs of RNA1 and RNA2 was named pPDV1&2-301. The presence of both fragments in pPDV1&2-301 was confirmed by Sanger sequencing and primer walking (Supplementary Table S1).

### 2.6. Agroinfiltration of Herbaceous Plants

The infectious clone constructs were transformed into *Agrobacterium tumefaciens* Eha105 [23] via electroporation [24]. *A. tumefaciens* cells harboring the infectious clone constructs were grown in Luria-Bertani medium containing kanamycin (100 µg mL^−1^) and rifampicin (20 µg mL^−1^) until they reached an optical density at 600 nm (OD_600_) of 0.5–0.7. *A. tumefaciens* cells were then harvested by centrifugation for five minutes at 3000× *g* and resuspended in agroinfiltration buffer (100 mM 2-(N-morpholino) ethanesulfonic acid pH 5.6, 100 mM MgCl_2_, 100 mM acetosyringone, 0.03% Tween 20, 20 μM 5 azacytidine and 0.5 mM ascorbic acid). The resuspended cells were then diluted to an OD_600_ of 1.0 and then mixed in a single culture tube immediately prior to infiltration. Approximately 200 μL of mixed cells were infiltrated into the abaxial side of both cotyledons of cucumber seedlings using a needleless syringe. Infiltration experiments were conducted at least three times and five plants were used for each treatment. For the infiltration of *N. benthamiana*, the fully expanded leaves of five-week-old plants were infiltrated with a needleless syringe. Fully expanded rosette leaves were infiltrated in experiments using *Arabidopsis thaliana*.

### 2.7. Agroinfiltration of Sweet Cherry

Cherry seedlings (approximately 10 to 12 days old) were agroinfiltrated essentially as described previously [25]. Whole plants were removed from soil and washed with sterile distilled water to remove remaining soil particles. The cotyledons and stem were wounded several times using a sterile 26-gauge needle. The wounded seedlings were submerged in a liquid mixture of *A. tumefaciens* strain EHA105 cultures harboring constructs pPDV1&2 301 and pPDV3-301which were grown in Luria-Bertani medium containing kanamycin (100 µg mL^−1^) and rifampicin (20 µg mL^−1^) until they reached an optical density at 600 nm (OD_600_) of 0.5–0.7 and then harvested by centrifugation for five minutes at 3000× *g* and resuspended in agroinfiltration buffer The resuspended cells were then diluted to an OD600 of 1.0 and then mixed in beaker. Once submerged, the wounded seedlings were subsequently subjected to vacuum infiltration for 5 min at −70 kPa in a vacuum chamber (Labconco Corporation, Kansas City, MO, USA). The pressure of the vacuum chamber was increased to ambient pressure over a period of 1 min. The infiltrated plants were then transplanted into new pots, covered with a transparent plastic cover to maintain high humidity for 48 h and were maintained using standard lighting and temperature conditions. Infiltration experiments using sweet cherry were conducted twice, with 5 seedlings receiving each treatment.

### 2.8. Mechanical Transmission

To test the transmissibility of PDV derived from the infectious clone, squash was used as herbaceous host. Approximately 10 g of symptomatic leaf tissue was collected from PDV infected cucumber and was ground with a mortar and pestle in 10 mL of mechanical inoculation buffer (0.01 M Na_2_H/KH_2_PO_4_ pH 7.2, 2% Polyvinylpyrrolidone MW 40,000, 1% 2-mercaptoethanol, 5 mM cysteine HCL, 1 mM Na diethyldithiocarbamate (Na-DIECA), 2 mM EDTA, 0.1% activated charcoal). Once homogenized, the cotyledons of squash plants were dusted with carborundum powder, and a gloved finger was dipped in the homogenate and gently rubbed on each cotyledon 5 times in a single direction with light pressure. Approximately 5 min after rubbing, extra inoculum was removed from cotyledons by light misting with tap water. Plants were covered to maintain high humidity for 48 h and then maintained in a growth chamber under standard plant growth conditions.

### 2.9. DAS-ELISA and RT-PCR

Detection of PDV after agroinfiltration or mechanical transmission was performed using DAS-ELISA, and RT-PCR. DAS-ELISA was performed using the PDV DAS-ELISA kit to detect the viral CP of PDV (Agdia, Elkhart, IN, USA) following the manufacturer’s specifications. Results from DAS-ELISA were analyzed using a Fisher Scientific BioTek Epoch 2 microplate reader (Thermo Fisher Scientific, Mississauga, ON, Canada). For RT-PCR total RNAs were extracted from newly emerging leaves. The extracted RNA was digested with DNAse I (Thermo Fisher Scientific, Mississauga, ON, Canada) and cDNAs were generated using the superscript III cDNA synthesis kit, utilizing random hexamer primers, following the manufacturers protocol (Thermo Fisher Scientific, Mississauga, ON, Canada). PCR was carried out with PDV specific primers which amplify a portion of the gene which encodes the CP (Supplementary Table S1). PCR reactions were comprised of 12 μL 2× Taq Froggamix (FroggaBio, Vaughan, ON, Canada), 1 μL of newly synthesized cDNAs, 1μL of each forward and reverse primer (each at a concentration of 0.2 μM), and 10 μL of sterile distilled water with a total volume of 25 μL. PCR was performed using an initial denaturation step of 3 min at 94°C, followed by 35 cycles of 30 s at 94 °C for denaturation, annealing for 30 s at 54 °C, and 30 s at 72°C for extension, followed by a 5 min extension step at 72 °C.

### 2.10. Transmission Electron Microscopy

A symptomatic leaf was placed in a 50 mm Petri dish and approximately 500 µL of extraction buffer (0.01 M Na_2_H/KH_2_PO_4_ pH 7.2, 0.005 M cysteine HCL, 0.01 M Na-DIECA) was placed on top of the leaf tissue. A glass rod was used to macerate the tissue in the buffer. Approximately 20 μL of the homogenate was placed on top of a 400 mesh formvar covered copper grid (Electron Microscopy Sciences, Hatfield, PA, USA) and incubated at room temperature for 5 min. The grid was then washed with 6 drops of ultrapure distilled water and dried by touching the edge of the grid with a piece of filter paper (Whatman 44 mm, Sigma Aldrich Canada, Oakville, ON, Canada). Negative staining was performed using 20 μL of 1% uranyl acetate for 1 min, the grid was subsequently dried with a piece of filter paper, and then allowed to air-dry at room temperature for 5 min. PDV virions were visualized using a Jeol JEM-1400Flash transmission electron microscope at 80 kV, at various magnifications (40,000×–80,000×).

## 3. Results

### 3.1. Identification of PDV via NGS

NGS of two sRNA libraries separately derived from symptomatic, and asymptomatic sweet cherry leaves (Figure 1) resulted in a total of 5,380,196 reads and subsequently 4,733,804 clean reads (17–27 nt long) after removal of the adapter sequence. These clean reads were assembled into contigs using Velvet Assembler 0.7.31 [19] and Oases 0.2.09 with k-mers of 17 [26]. BLASTn analysis using the assembled contigs identified four viruses including PDV, PNRSV, Cherry virus A (CVA), and Little cherry virus 1 (LChV1). As PDV is an important viral pathogen of *Prunus* species that may be associated with the *Prunus* fruit decline, we chose PDV for further study.

We surveyed the PDV incidence in an orchard plot by detection of the virus using DAS-ELISA. Among 92 trees, 39 were positive. The PDV incidence was calculated to be 42.4%. Since protein-based detection is usually less sensitive than RT-PCR, we also isolated total RNA from half of the 92 trees by selecting alternating trees and conducted RT-PCR using a pair of primers for PDV detection (Supplementary Table S1). We found that half of the samples (50%) were PDV positive. These results suggest that PDV is indeed a widespread virus with high incidence in the plot examined.

### 3.2. Cloning and Sequencing of the Complete Genome of PDV

We further cloned and determined the full-length sequence of all three RNA fragments (the complete viral genome of PDV) by RT-PCR, RACE-PCR and primer walking. The genomic sequences of the three RNAs were deposited in GenBank with accession nos. MK522387, MK522388 and MK560342. The sequence of RNA1 is 3374 nt in length and shares 96% sequence identity with an isolate identified in Slovakia (GenBank accession No. MF078478). This genomic RNA has a 5′ UTR of 38 nt and has a single ORF which is 3168 nt long encoding the P1 protein. Sequence analysis using the HHpred server [27] indicates P1 contains a both methyltransferase and helicase domains (Table 1). The 3′ UTR of RNA1 is 169 nt in length and possesses four AUGC repeats which are predicted to form complex secondary structures involved in the viral infection cycle [9]. The RNA2 of PDV is 2596 nt in length with 95% sequence identity to an isolate of PDV identified in the United States (GenBank accession No. AF277662). This RNA has a 5′ UTR that is 33 nt in length, and a single ORF which is 2367 nt long, encoding the P2 protein. A domain with characteristics of a viral RdRp (Table 1) was identified, when the obtained sequence of the RNA2 ORF was analyzed using HHpred [27]. The 3′ UTR of this RNA is 192 nt in length with a series of four AUGC repeats, similar to the first RNA fragment. The third and shortest RNA fragment 2296 nt in length sharing 91% sequence similarity with an isolate sequenced in the United States (GenBank accession No. L28145). The 5′ UTR of RNA3 is 428 nt in length. ORF3a, which encodes the viral MP has a length of 879 nt. The RNA3 also contains a region downstream of ORF3a with a high A/T composition (66%). This region resembles a subgenomic promoter to allow for the transcription of a sgRNA molecule sgRNA4 which is 986 nt long and codes for ORF3b. This 654 nt ORF encodes the CP, in which the highly conserved RNA binding motif K_10_P_11_T_12_A_13_R_14_S_15_Q_16_N_17_F_18_A_19_ was easily identified. While some ilarviruses have a second RNA binding motif in their CPs, this motif was not found in the CP sequence reported in this work. RNA3 and sgRNA4 share the same 3′ UTR which is 257 nt long and like RNA1 and RNA2, this UTR contains 4 AUGC repeats likely permitting the formation of alternate RNA structures.

### 3.3. Construction of the Full-Length cDNA Clone of PDV

**Figure 2:**
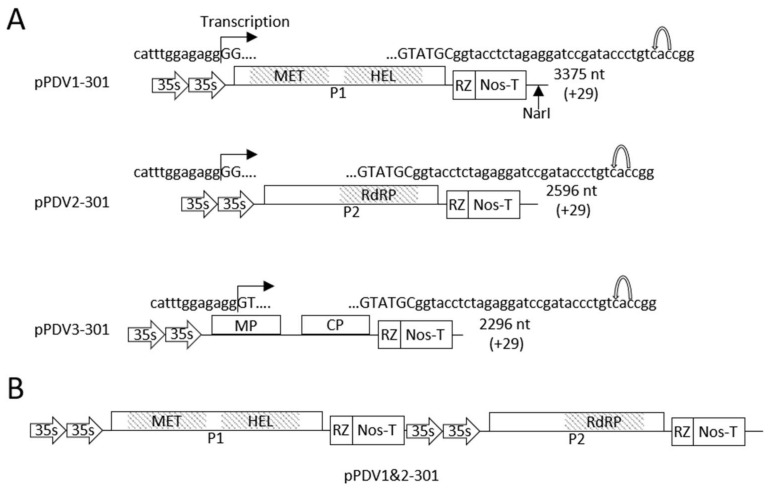
Schematic diagram of the infectious clone of PDV for in plant studies. (**A**) Tripartite infectious clone of PDV is comprised of cDNAs of each PDV genomic RNA separately inserted into the vector pCB-Rz. (**B**) cDNAs of PDV genomic RNA fragments 1 and 2 were combined into a single vector to increase the infectivity rate of the PDV infectious clone. Single black lines and lined boxes represent noncoding and coding regions of each RNA fragment, respectively. The protein encoded by each coding region is labelled: P1 (replicase), P2 (RdRp), MP and CP. Identified functional domains of proteins are shown as grey boxes. Transcription start sites are shown at the 5′ end of each construct by a bent arrow following the promoter (arrows containing “35 s”) sequence. At the 3′ sequence the uppercase and lowercase letters represent the 3′ sequence of viral RNA and the non-viral sequence of the hammerhead ribozyme (box containing “RZ”), respectively. The bent arrow at the 3′ end indicates the self-cleavage site of the ribozyme. The nucleotide length of RNA1, RNA2 and RNA3 is shown to the right with the number of additional nucleotides after ribozyme self-cleavage provided in parentheses. The single *Nar*I restriction enzyme recognition sequence is shown in pPDV1-301 which was used for the integration of the cDNA corresponding to the viral genomic RNA2 resulting in the construction of pPDV1&2-301.

The full length cDNAs of three PDV RNA fragments were separately cloned into the vector pCB-Rz (Figure 2A) between the transcription regulatory elements (the double 35s promoter and the hammerhead ribozyme), to generate pPDV1-301, pPDV2-301 and pPDV3-301. Because both the 5′ and 3′ termini of ilarviruses and thus PDV are suspected to be involved in processes such as viral replication and virion encapsidation, the entire genomic sequences were included in the design of this infectious clone, not simply the protein coding sequences. Primers used to amplify the cDNAs of genomic RNAs were specifically designed so the transcription start site beginning at the first nucleotide “G” of all three genomic cDNAs, to produce authentic viral 5′ untranslated regions. The transcription of the viral genome was halted by the self-cleaving ribozyme, which allows for the entire viral genome to be transcribed with the addition of 29 non-viral nucleotides at the 3′ end (Figure 2A). A previous study with a PNRSV infectious clone has shown that combining cDNAs of RNA1 and RNA2 into a single vector greatly improved the infection rate compared to using three separate constructs [22]. The cDNA of RNA2, and the transcription regulatory cassette was amplified from pPDV2-301 with primers containing flanking *Nar*I restriction enzyme recognition sites and was successfully cloned into pPDV1-301 to generate pPDV1&2-301, a single construct containing both cDNAs of viral RNA1 and RNA2 (Figure 2B).

### 3.4. The Full-Length cDNA Clone of PDV Is Infectious on the Natural Host Sweet Cherry

**Figure 3:**
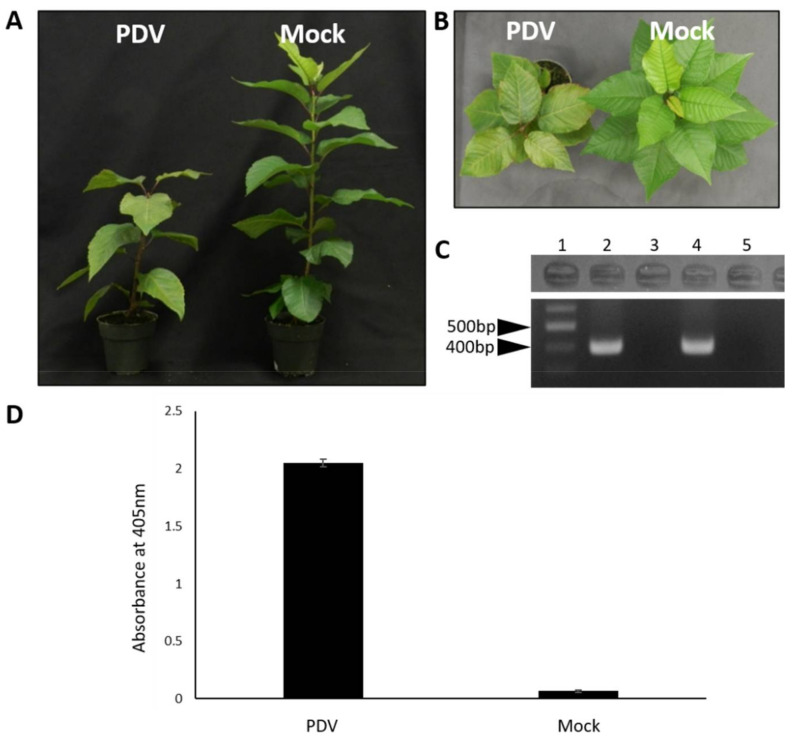
The PDV infectious clone is infectious on its natural host sweet cherry. Seedlings of cherry were agroinfiltrated with the PDV infectious clone to test the infectivity of this construct on the natural host. (**A**) Side view of seedlings infiltrated with the PDV infectious clone and empty vector (mock). Seedlings infected by PDV (left) have shorter internodal lengths resulting in a dwarfed or stunted phenotype compared to mock treated plants (right). (**B**) Aerial view of seedlings infiltrated with the PDV infectious clone or empty vector (mock). PDV infected seedlings (left) produce fewer leaves compared to mock treated plants (right). (**C**) The presence of PDV in upper non-infiltrated leaves of dwarfed cherry plants (left) was detected by RT-PCR. Lane 1: 1000 bp DNA ladder; 2: non infiltrated distal leaf of PDV infected cherry seedling at 8 wpa; 3: distal leaf of mock treated plant at 8 wpa; 4: a sample of foliar tissue known to be infected with PDV served as a positive control; 5: water was used as a negative control to test PCR reactions. (**D**) DAS-ELISA confirmed the presence of PDV in the distal, non-infiltrated leaf samples of the seedlings agroinfiltrated with the PDV infectious clone. The absence of PDV was confirmed in mock treated plants. The error bars represent the standard deviation of the means (*n* = 5). This experiment was performed twice, and each experiment consisted of 5 seedlings receiving each treatment.

To test the infectivity of the PDV full-length cDNA clone on natural hosts, pPDV1&2_301 and pPDV3_301 were agroinfiltrated into seedlings of sweet cherry. Prior to agroinfiltration, RT-PCR analysis using PDV CP specific detection primers (Supplementary Table S1) confirmed that that these seedlings were free from PDV. Approximately 8 weeks post agroinfiltration (wpa), 60% of the seedlings agroinfiltrated with the PDV full-length cDNA clone showed severe stunted growth with shorter stem and fewer leaves but without obvious foliar symptoms compared to mock-infiltrated plants (Figure 3A,B). RT-PCR and DAS-ELISA performed on distal, non-infiltrated leaves of these seedlings confirmed the presence and long-distance movement of PDV in symptomatic trees, and absence of PDV in mock-infiltrated seedlings (Figure 3C,D). These data suggest that the PDV full-length cDNA clone generated here is infectious on its natural host.

### 3.5. The Full-Length cDNA Clone of PDV Does Not Infect Arabidopsis

**Figure 4:**
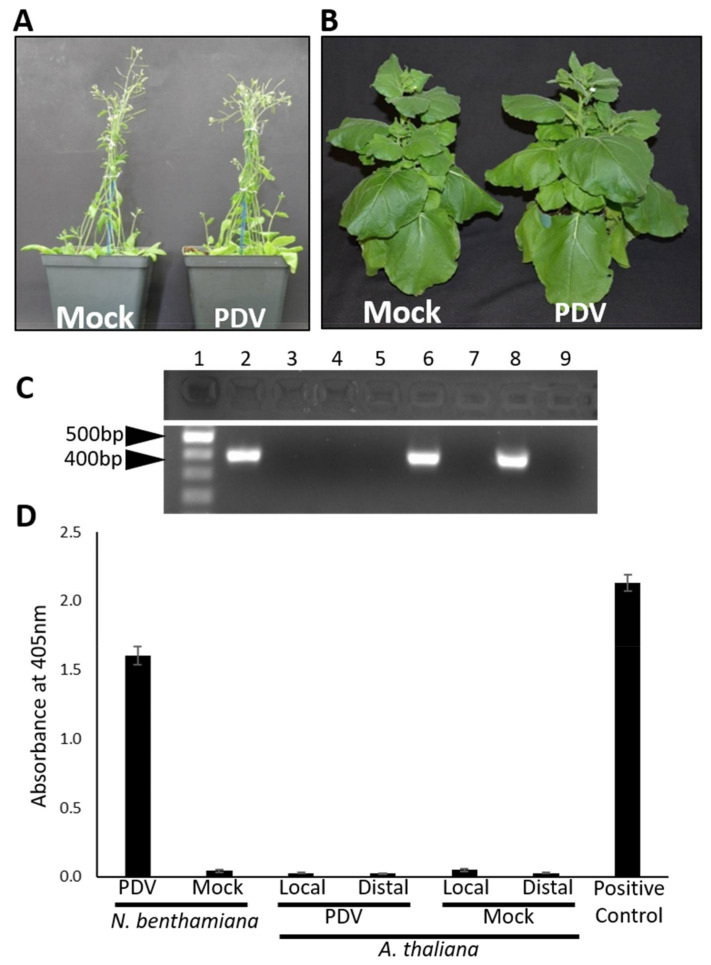
PDV does not infect Arabidopsis and infects *N. benthamiana* latently. The cDNA clone of PDV was used to infiltrate commonly used experimental host plants. (**A**) Arabidopsis seedlings agroinfiltrated with the full-length cDNA clone of PDV (right) did not develop any visible symptoms and could not be differentiated from mock inoculated plants (left) at 21 dpa. (**B**) *N. benthamiana* plants agroinfiltrated with the full-length cDNA clone of PDV did not develop any visible symptoms and could not be differentiated from mock treated plants at 21 dpa. (**C**) RT-PCR was used to detect PDV in both plants. The coding sequence of the CP was amplified as 900 bp in size. Lane 1: 1000 bp DNA ladder; 2: locally infiltrated Arabidopsis rosette leaf at 7 dpa; 3: distal leaf of PDV infiltrated Arabidopsis at 21 dpa; 4: mock infiltrated rosette leaf of Arabidopsis; 5 distal leaf of mock infiltrated plant; 6: upper leaf of PDV infiltrated *N. benthamiana* plant at 21 dpa; 7: upper leaf of mock infiltrated *N. benthamiana* plant at 21 dpa; 8: A sample of foliar tissue known to be infected with PDV served as a positive control; 9: water was used as a negative control to test PCR reactions. (**D**) Relative levels of PDV accumulation in locally infiltrated leaves of Arabidopsis at 7 dpa and distal leaf tissues of Arabidopsis and *N. benthamiana* at 21 dpa were determined by DAS-ELISA. Error bars represent the standard error of the means. Arabidopsis plants were maintained for a total of 5 weeks after infiltration, studies on Arabidopsis were performed as 3 separate experiments consisting of 12 Arabidopsis seedlings receiving each treatment per experiment. *N. benthamiana* plants were maintained for a total of 5 weeks after infiltration and studies on *N. benthamiana* were performed as 3 separate experiments. During each experiment, 12 *N. benthamiana* seedlings received each treatment. Mock inoculated Arabidopsis and *N. benthamiana* plants did not generate PDV specific amplicons by RT-PCR nor were positive results obtained by DAS-ELISA from these plants.

To study the viral infection cycle, and molecular virus–host interactions for the development of novel antiviral technologies, model plants are commonly used as they have a short growing cycle and are amenable to laboratory techniques such as genetic transformation [28]. The most used model plants in plant virology are Arabidopsis and *Nicotiana benthamiana* [28,29]. To test the infectivity of the PDV clone, rosette leaves of Arabidopsis were agroinfiltrated with an equal mixture of three cultures of *A. tumefaciens* strain EHA105 each harboring PDV1_301, PDV2_301, and PDV3_301. Plants were closely monitored for the development of symptoms. At 7 days post agroinfiltration (dpa), samples of the infiltrated rosette leaves were collected and frozen. At 21 dpa no disease symptoms were observed. Plants agroinfiltrated with the infectious clone could not be distinguished from plants agroinfiltrated with the empty plasmid vector pCB-Rz (mock treatment; Figure 4A). At 21 dpa, distal new leaves of the plants agroinfiltrated with the full-length cDNA clone or empty vector were collected. All tissue samples were analyzed by RT-PCR and DAS-ELISA to determine if the plants were infected by PDV. RT-PCR to detect the CP of PDV resulted in small amounts of amplicons of correct size in infiltrated Arabidopsis leaves but not in distal leaves of the same plant, nor in the mock treated plants (Figure 4C). DAS-ELISA failed to detect the viral CP of PDV in all plant tissues tested including locally infiltrated and distal tissues of PDV infiltrated, and mock inoculated plants (Figure 4D). These results suggest that the full-length cDNA clone of PDV is not infectious on Arabidopsis.

### 3.6. The Full-Length cDNA Clone of PDV Infects N. benthamiana Asymptomatically

To determine if *N. benthamiana* could be infected by the cDNA clone of PDV, fully expanded leaves of this model plant were infiltrated with an equal mixture of three cultures of *A. tumefaciens* each harboring the T-DNA construct pPDV1_301, pPDV2_301 and pPDV3_301. At 21 dpa, all plants appeared healthy showing no disease symptoms as PDV and mock infiltrated plants could not be visually differentiated (Figure 4C). When RT-PCR and DAS-ELISA were performed to detect PDV, positive results for both tests were found in the distal leaf tissues of *N. benthamiana* agroinfiltrated with the full-length cDNA clone. RT-PCR did not detect PDV in mock infiltrated plants, and the absorbance reading was insignificant when DAS-ELISA was performed. Initial studies showed the infectious clone had a low average infectivity rate of 30% (3 out of 10 infiltrated plants). Co-infiltration with the mixed *A. tumefaciens* cultures separately harboring the constructs pPDV1&2_301 and pPDV3_301 (Figure 2) achieved a higher average rate of infection of 90 to 100% (data not shown). These data suggest that the full-length cDNA of PDV is infectious on *N. benthamiana* where PDV infection is latent under given growth conditions.

### 3.7. The Full-Length clone of PDV Infects Cucumber

**Figure 5:**
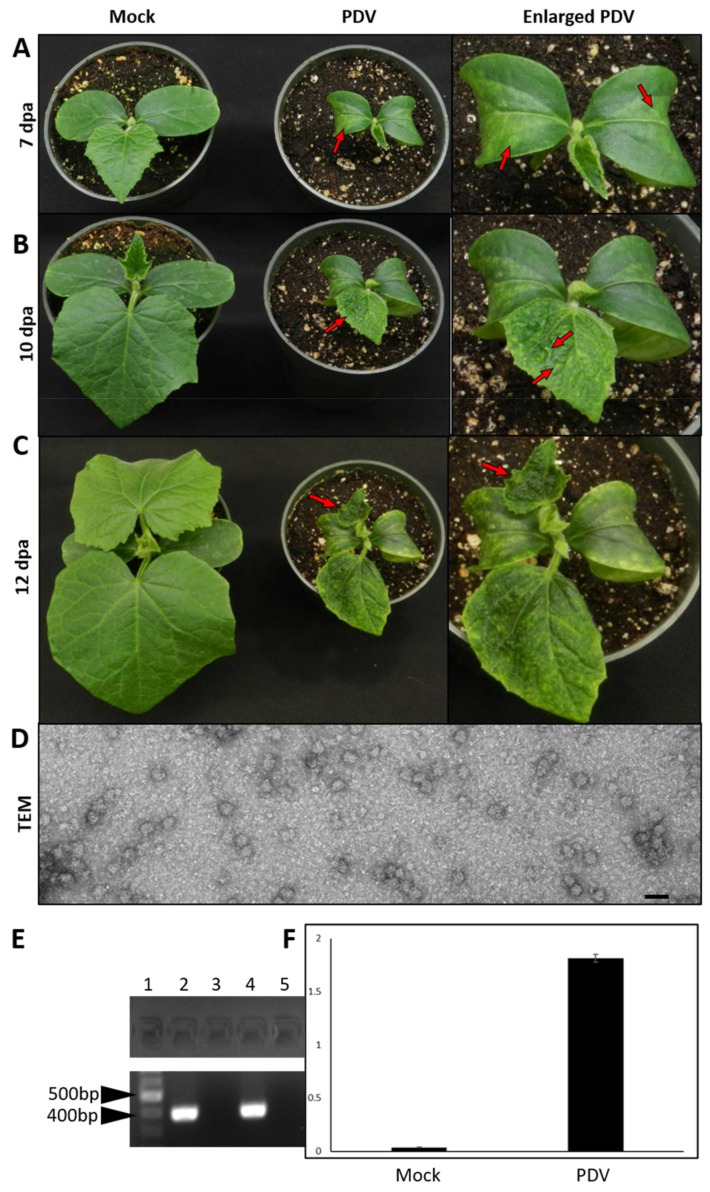
Cucumber serves as a symptomatic host of PDV. To evaluate the use of cucumber as an experimental host for PDV infection, cotyledons of cucumber cv. ‘Wisconsin’ were agroinfiltrated with the full-length cDNA clone of PDV. Left, mock-inoculated; middle, agroinfiltrated with the PDV infectious clone; right, enlarged true leaf of the middle plant. (**A**) At 7 dpa, PDV symptoms are visible on the newly emerging first true leaves as small chlorotic spots and some vein clearing (red arrows). (**B**) At 10 dpa chlorotic leaf spotting is present on the fully expanded first true leaf (red arrows). (**C**) At 12 dpa the second true leaf of PDV infected cucumber exhibits stronger symptoms including chlorosis and leaf deformation (red arrows). (**D**) Isometric shaped virions isolated from PDV infected cucumber leaves were visualized by transmission electron microscopy. (**E**) The presence of PDV in upper non-inoculated leaves of symptomatic cucumber plants was detected by RT-PCR. Lane 1: 1000 bp DNA ladder; 2: non infiltrated first true leaf of PDV infected plant at 12 dpa; 3: distal leaf of mock treated plant at 12 dpa; 4: A sample of foliar tissue known to be infected with PDV served as a positive control; 5: water was used as a negative control to test PCR reactions. (**F**) Relative levels of PDV accumulation in upper, non-infiltrated leaves were determined by DAS-ELISA. Error bars represent the standard deviation of the mean of 9 seedlings for each treatment. This study was performed in 3 independent experiments consisting of 9 cucumber seedlings receiving each treatment during each experiment. For all studies, mock inoculated plants did not generate PDV specific amplicons nor positive results by DAS-ELISA.

Cucumber has previously been used as an indicator host when indexing woody hosts infected with PDV [30]. More recently, cucumber has served as an experimental host for PNRSV [17]. To test if cucumber would serve as a more appropriate host to study processes such as disease symptom development, fully expanded cotyledons of cucumber were agroinfiltrated with the bipartite PDV cDNA clone. When plants were closely examined, PDV agroinfiltrated plants showed disease symptoms and these plants were easily differentiated from mock infiltrated plants (Figure 5A–C). The symptoms on cucumber progressed over time and increased in severity. At 7 dpa, chlorotic lesions were visible on agroinfiltrated cotyledons of PDV infected plants (Figure 5A, red arrows) at 10 dpa first true leaves were deformed in shape, displayed chlorotic leaf spots, and had sunken first order veins (Figure 5B, red arrows). At 12 dpa, PDV infected cucumber plants were obviously dwarfed in stature when compared to mock treated plants, additionally, newly emerging second true leaves also showed severe leaf deformation and mottled symptoms (Figure 5C). Plants which were mock inoculated with *A. tumefaciens* harboring the empty vector pCB-Rz did not display any symptoms at any time points (Figure 5A–C). When symptomatic cucumber leaf tissues were prepared for visualization by electron microscopy, isometric PDV virions were visible and ranged in size from 25–30 nm in diameter (Figure 5D). To confirm that these symptoms were caused by PDV infection, RT-PCR and DAS-ELISA were used and symptomatic plants were indeed positive for PDV using both tests (Figure 5E,F).

### 3.8. PDV Derived from the Infectious Clone Transmits Mechanically

**Figure 6:**
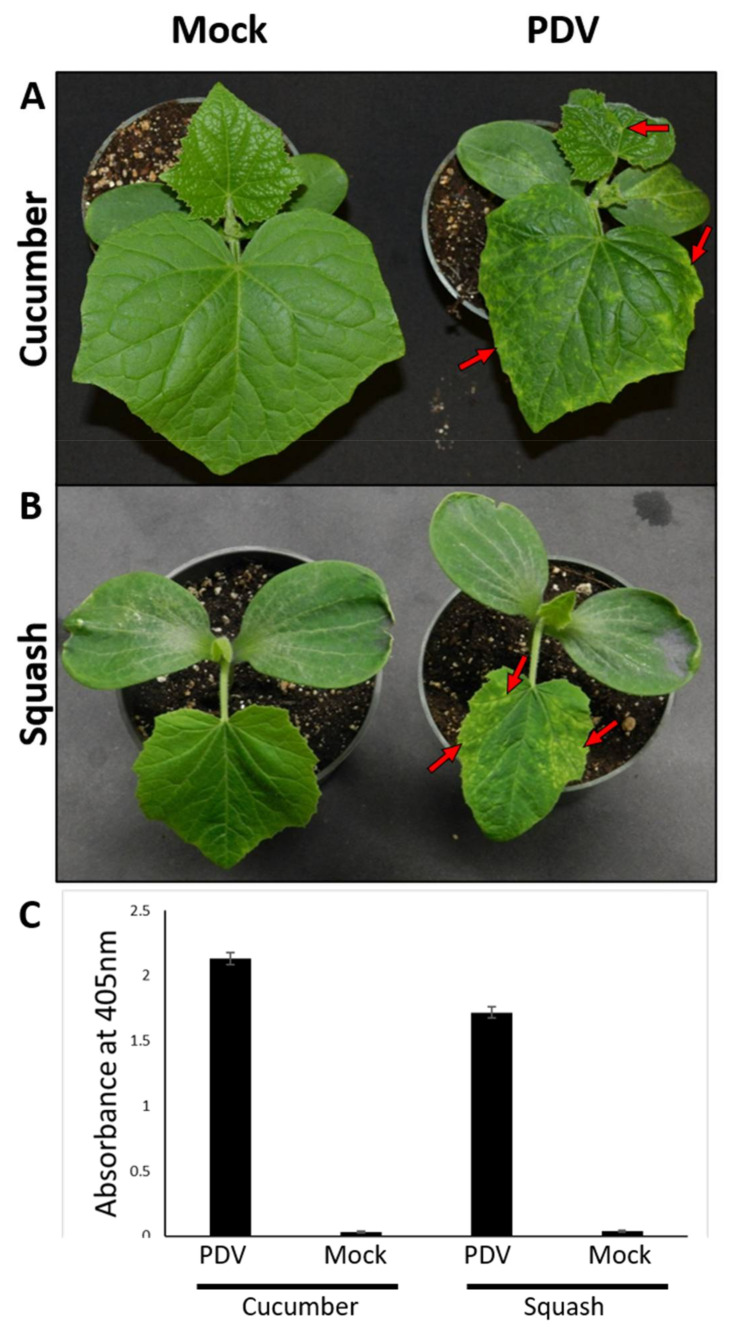
PDV derived from the cDNA clone is mechanically transmissible. The cotyledons of squash and cucumber seedlings were mechanically inoculated with leaf tissues from healthy control plants or with the symptomatic leaves of PDV infected cucumber plants. (**A**) PDV symptoms are clearly seen as chlorotic leaf spots at 9 dpi (red arrows). (**B**) When squash seedlings were mechanically inoculated at 9 dpi chlorotic spots were seen at the margins of the first true leaves (red arrows). (**C**) DAS-ELISA was used to confirm PDV infection and was used to compare relative abundance of PDV at nine dpi. Error bars represent the standard deviation of the mean. Studies on mechanical transmission were performed as three independent experiments each consisting of 5 seedlings of each species for each treatment during each experiment. In all experiments, no positive results were obtained by DAS-ELISA from mock inoculated plants.

A classical method of inoculation for studying of viruses is mechanical inoculation, which predates Agrobacterium-mediated inoculation but is still widely used today [30,31]. To test if PDV derived from this infectious clone could be transmitted via mechanical inoculation symptomatic tissues of PDV infected cucumber plants (previously agroinfiltrated with the PDV infectious clone) were used as inoculum to rub-inoculate healthy cucumber and squash plants. At 9 days post inoculation (dpi) symptoms like those observed in agroinfiltrated cucumber plants were observed in mechanically inoculated cucumber seedlings (Figure 6A). Additionally, symptoms developed on the first true leaves of mechanically inoculated squash seen as chlorotic spots on the leaf margins (Figure 6B). For both cucumber and squash, mock inoculated plants did not display any symptoms. DAS-ELISA was used to confirm PDV was present in symptomatic plants, and absence of PDV in mock inoculated plants (Figure 6C).

Considering the pathogenicity tests on herbaceous hosts, it appears that Arabidopsis is not susceptible to the PDV infectious clone, *N. benthamiana* may serve as an asymptomatic host and lastly, and both cucumber and squash are symptomatic hosts of PDV. Therefore, the two cucurbit species are suitable for the molecular studies of the PDV infection process such as, viral replication and movement, disease development and pathogenicity, and molecular PDV–plant interactions.

## 4. Discussion

As a member of the *Ilarvirus* genus, PDV is an important viral pathogen that impacts global stone fruit production [3]. In sweet and sour cherry, PDV and PNRSV are the most common viruses of these crops [32]. Despite its danger to stone fruit production and prevalence in stone fruit growing regions, PDV is still a relatively understudied virus [4]. This is at least partially attributed to the unavailability of a PDV infectious clone. Infectious cDNA clones of many plant viruses, particularly those herbaceous plant-infecting viruses, have been constructed and serve as a powerful tool for reverse genetic studies on these dangerous pathogens [16,33,34]. Additionally, these infectious clones have been utilized as gene expression systems to confer virus resistance to hosts by overexpression or silencing of host genes [22,35]. However, it is usually very challenging to develop infectious clones for woody plant-infecting viruses.

In this study, the first full-length infectious clone of PDV was constructed with transcription being driven by CaMV 35s promoters (Figure 2). Firstly, the genomic sequence of an isolate of PDV was determined. This isolate was associated with severe foliar symptoms on sweet cherry within the Niagara Fruit belt, a significant stone fruit producing region in Ontario, Canada. This sequenced isolate served as the source of the cDNA clone which was constructed. The ability to introduce this clone by agroinfiltration in both herbaceous and woody hosts provides a simple and inexpensive method to study processes related to viral infection in species traditionally recalcitrant to agrobacterium mediated methods [36]. We verified that this clone is indeed infectious on the natural host, cherry, and causes a dwarfing phenotype of infected seedlings (Figure 3A,B). The use of agroinfiltration to deliver infectious clones is simpler, and more cost effective compared to other systems such as biolistic bombardment. While this method has been used successfully in apple seedlings [25] agroinfiltration has not been performed in stone fruit seedlings such as cherry which was reported in this work (Figure 3). By using agroinfiltration to infect cherry seedlings with this infectious clone, studies on PDV may become accessible to more laboratories given the low initial costs of this system. Time and spatial constraints are two major obstacles in the study of perennial hosts such as fruit trees, and thus a smaller, faster growing host is more advantageous to studying PDV in the laboratory.

The inability of PDV to infect *Arabidopsis* (Figure 4) is also the case for PNRSV [37], suggesting this plant is not suitable for studying PDV infection processes. *N. benthamiana* has long been used as a host to study plant virus infection as this plant is seemingly hypersusceptible to many viruses [38]. The observation that PDV infection on *N. benthamiana* does not induce obvious symptoms is similar to infection by PNRSV [22]. The lack of disease symptoms on this model plant may be advantageous for studying some processes of the viral infection cycle such as viral replication. However, disease progression and identification of determinants of pathogenicity is better studied in a symptomatic host plant. Cucumber serves as a suitable experimental host to study PDV infection as this host reacted strongly to infection, displaying a stunted phenotype and foliar symptoms (Figure 5A–C). Cucumber presents several advantages for laboratory research as this plant grows quickly, and requires less space compared to fruit trees.

The ability of PDV to infect *N. benthamiana*, cucumber and cherry suggests these species possess similar host factors which can be usurped by PDV for viral replication and systemic movement [39]. It is possible that host factors required for PDV infection are either absent in Arabidopsis, or antiviral defense mechanisms such as non-host resistance or unknown resistance genes prevent a compatible interaction between PDV and this host. The underlying mechanisms are certainly worth further investigation.

When comparing symptom development of cherry and cucumber, the ability of PDV to cause dwarfing phenotypes in both hosts suggests that similar biological pathways are impacted during viral infection. The discrepancy in foliar symptoms between these two hosts likely results from specific molecular interactions between PDV and these two plant species. Persistent viral infection in perennial hosts is often associated with low viral titer and in some cases, reduced virulence is seen even at higher viral concentrations [40]. Since cherry is a long-lived perennial woody host, the lack of foliar symptoms may be related to a lower accumulation of PDV, or a reduction in virulence in this host. Ilarviruses such as PDV are known to persist at low titers in their natural woody hosts, and in fact, false negative diagnosis of ilarviruses in *Prunus* species is often attributed to their low titer [6]. In contrast to the apparent absence of foliar symptoms on cherry, the severe leaf symptoms found on infected cucumber plants may be a consequence of PDV accumulating to higher levels in this faster-growing annual herbaceous host.

In summary, this work presents the generation of the first full-length infectious clone of PDV, demonstrating its infectivity in natural and experimental hosts. The availability of this infectious clone makes it possible to study PDV pathogenesis, synergic interactions with other viruses and diverse aspects of PDV such as viral replication and intercellular movement and molecular PDV–host interaction, which ultimately facilitates the development of novel strategies to control PDV and related viruses.

## Figures and Tables

**Table 1:** Regions of the sequenced PDV genome resembling functional domains.

nt Position.	Description ^a^	Protein ID ^b^	HHpred Hit	Probability (%)	E-Value ^c^
PDV RNA 1					
1–38	5’UTR	-	-	-	-
39–3206	P1	QGA70955.1	-	-	-
-	Rep	-	3VKW_A	100	3.20 × 10^−42^
-	Met	-	PF01660.17	100	3.10 × 10^−35^
-	Hel	-	6JIM_B	99.91	1.40 × 10^−25^
-	DNA Binding	-	4B3F_X	99.89	3.10 × 10^−25^
3207–3375	3’UTR	-	-	-	-
PDV RNA 2					
1–33	5’UTR	-	-	-	-
34–2400	P2	QGA70956.1	-	-	-
-	RdRp	-	PF00978.21	100	2.20 × 10^−52^
2401–2593	3’UTR	-	-	-	-
PDV RNA 3					
1–428	5’UTR	-	-	-	-
429–1310	MP	QGA72060.1	PF01573.16	100	1.60 × 10^−75^
1382–2038	CP	QGA72061.1	PF01787.16	100	5.00 × 10^−65^
2039–2296	3’UTR	-	-	-	-

^a^ UTR: untranslated region, Rep: replicase domain, Met: methyltransferase domain, Hel: helicase domain, RdRp: RNA dependent RNA polymerase, MP: movement protein, CP: coat protein. ^b^ Genbank protein identifiers were used in this study. ^c^ E-value: expected number of false positives per database search which scores the same or better than the sequence percent identity.

## Data Availability

The data presented in this study are available on request from the corresponding author.

## Supplementary Materials

The following are available online at https://www.mdpi.com/article/10.3390/v13102025/s1, Table S1: Primers used for the detection, complete genome amplification and infectious clone construction of PDV.
