# Supplementary material for: Molecular Identification of Prune Dwarf Virus (PDV) Infecting Sweet Cherry in Canada and Development of a PDV Full-Length Infectious cDNA Clone
Source: Viruses. 2021 Oct 7;13(10):2025. doi: 10.3390/v13102025 (PMC8541084; doi:10.3390/v13102025)
Supplement: Supplementary file 1 [file viruses-13-02025-s001.zip › viruses-1412231-supplementary.pdf]

Supplementary Table S1. Primers used for the detection, complete genome amplification and infectious clone construction of PDV.

Primer name[a]	Sequence (5' - 3')[b]	Amplicon size (bp)[c]	Melting temperature (°C)
	**PDV Genome walking primers**		
PDV1sqF1	GATGAGACCACTACCGTCGG	849	60
PDV1sqR1	CAACTCAATCCCAGGTTTATACCCG		59
PDV1sqF2	CAATGCTGTTGATCTAGGTGATGCCG	883	62
PDV1sqR2	CTTAGAAGCAACAAGGGCACGC		60
PDV1sqF3	GATTCTTAACGGGATACATGGAAGTCG	1049	59
PDV1sqR3	GATTGTAGCAGCAGCATATATGCACCC		62
PDV1sqF4	CTCTTGACTTCCAACAGATCTTCTGC	665	59
PDV1sqR4	GATAAAGCCACAAGTCCATGGCAAGC		62
PDV2sqF1	GTTTTTCGGATTTCTTATGATCCGAG	1046	56
PDV2sqR1	CTTCACCAATGGAAATAACGTCTGGC		60
PDV2sqF2	CATTCTCAACACTGTATTGAATGTCG	919	56
PDV2sqR2	GTTCGGTACTGCAACAACTTGC		58
PDV2sqF3	CAAACGTTGCTATGGTGGTTGC	604	59
PDV2sqR3	CAAAATAGATAGTATGGTCATCCACCG		57
PDV3sqF1	GTGAAACAATTCTGTGACGTTCG	949	56
PDV3sqR1	CTTTAAGAGGAACAGACTCGGC		56
PDV3sqF2	GATACTCCAGACATTTGCCAAATCACG	905	60
PDV3sqR2	GAATCAGGGATTTCGACTCTCTTAGGC		60
	**PDV 5' and 3' RACE primers**		
PDV1-5prGSP1	CTTGGACTCATCATGTAGTCCG	-	56
PDV1-5prGSP1	CAAAAGTTCATGGACATTGGCAGC	-	59
PDV1-3prGSP1	CATGAGGTTCAAGGAGGTAGC	-	56
PDV1-3prGSP1	GCTTTATCAAGACACAAGTCGG	-	55
PDV2-5prGSP1	GTTGAGCATCAGTAAGTTCAAGATCG	-	58
PDV2-5prGSP1	GAAAAACTGGCACAACGACCTGC	-	61
PDV2-3prGSP1	CTTCTCAGTTTTTGGTTTCGGC	-	56
PDV2-3prGSP1	GAGATAAAAATCCAGATTTACCCATGC	-	56
PDV3-5prGSP1	CAATTCAGGAGATAAATCTACGACCG	-	57
PDV3-5prGSP2	CTTCACACTTTGCAAGAAACCTTGC	-	59
PDV3-3prGSP1	CTTGCTCCTACTGACATGACCG	-	58
PDV3-3prGSP2	GAAATATTCGTAGTTGGAGATGCTGC	-	58
	**PDV detection primers**		
PDVcdetF	CGGACCAAATGTACCCGTGA	490	60
PDVcpdetR	TACAGAACCACACCGGAACG		59
	**Infectious clone primers**		
PDV1-PromF	*AAGGAAGTTCATTTCATTTGGAGAGG***GGTTTTACGAACGTGGTTGTTCG**	3428	58

PDV1-RZR	*CAGGGTATCGGATCCTCTAGAGGTACC***GCATACCTTAAAGGGGCATCCTCAC**		61
PDV2-PromF	*AAGGAAGTTCATTTCATTTGGAGAGG***GGTTTTACGAGCGTGGTTGTTCG**	2649	60
PDV2-RZR	*CAGGGTATCGGATCCTCTAGAGGTACC***GCATCCCTTAAAGGGGCATCC**		59
PDV3-PromF	*GAAGTTCATTTCATTTGGAGAGG***GTTTTTATAATCAAGAGAACTGAATAAATTG**	2346	54
PDV3-RZR	*CAGGGTATCGGATCCTCTAGAGGTACC***GCATCCCTTAAAGGGGCATCC**		59
R2NarIF	TATATAGGCGCCCTCGCATGCCTGCAGGTCAACATGGTGGAGC	3416	62
R2NarIR	ATATATGGCGCCCGATCTAGTAACATAGATGACACCGCGC		60

[a] F: forward primer, R: reverse primer, sq: sequencing primer, PDV: Prune Dwarf Virus, det: detection, 5pr: 5' RACE, 3pr: 3' RACE, GSP: gene specific primer for RACE-PCR, Prom: homology with 35s promoter, RZ: homology with ribozyme, NarI: primer contains NarI restriction enzyme site.

[b] Bolded: viral genome or viral protein coding sequence, Underlined: NarI restriction enzyme site, Italics: sequence homology to vector PCB301-d35sRZT

[c] Amplicon sizes of RACE primers are unknown as viral 5' and 3' ends are variable
